# A Study on the Radiation Cooling Characteristics of *Cerambycini Latreille*

**DOI:** 10.3390/biomimetics9010034

**Published:** 2024-01-04

**Authors:** Jie Xu, Delei Liu

**Affiliations:** 1 College of Modern Agriculture, Changchun Polytechnic, Changchun 130022, China; 2 School of Bionic Engineering, Jilin University, Changchun 130022, China; ldl20@mails.jlu.edu.cn

**Keywords:** radiation cooling, cerambycini latreille, bionic, structure functional surface, effective medium theory, alkaline etching

## Abstract

The severe climate and energy issues require more environmentally friendly and efficient cooling methods. Radiative cooling offers a cooling solution with significant advantages. However, current radiative cooling technologies focus primarily on seeking perfect materials to achieve complete wavelength absorption. However, numerous research studies have shown that achieving such a perfect scenario is not feasible. Here, inspired by the surface of the *Cerambycini Latreille*, the inherent mechanism of radiative cooling functionality in the unique structure of these hairs is revealed using effective medium theory and Finite Difference Time Domain (FDTD) optical simulation analysis. Through alkaline etching and template methods, a biomimetic radiative cooling film (BRCF) was successfully fabricated. The BRCF not only efficiently reflects solar radiation but also enhances absorption in the atmospheric window wavelength range. The radiative cooling mechanism proposed in this study and the BRCF presented here may inspire researchers to further explore the field of structural radiative cooling.

## 1. Introduction

Currently, climate issues are becoming increasingly severe, and the impacts of global warming-induced extreme weather are already affecting people’s lives [1,2,3,4]. Moreover, with the rapid pace of urbanization, phenomena such as population growth, anthropogenic heat emissions, and changes in land cover will lead to more pronounced urban heat island effects [5,6,7,8]. In summary, the current pressing climate and energy issues demand more environmentally friendly and efficient cooling methods [9,10]. In addition to developing clean energy sources such as solar, wind, and tidal energy, altering temperature regulation methods is also an ideal approach. Radiative cooling, being a method that requires no additional energy consumption and produces no greenhouse gases, stands out as a favorable cooling strategy. In the early stages of radiative cooling research, the researchers’ primary focus was on finding a material that could be treated as a blackbody in the 8–13 μm wavelength range [11]. This material should achieve complete absorption within this range and also achieve a complete reflection of visible light and near infrared, a capability known as spectral selectivity [12]. However, numerous research studies have shown that achieving such a perfect scenario could be not feasible. Currently, commonly used materials include silicon compounds [13,14], certain metallic compounds [15,16,17], and organic polymers [18,19,20,21,22], among others. However, research outcomes indicate that this approach cannot achieve radiative cooling perfectly. Therefore, the search for a new type of radiative cooling material is urgent.

In the natural world, many insects possess the ability to survive in hot environments, thanks to their unique body structures [23,24,25,26,27]. They have intricated micro- and nano-scale structures that not only increase the heat dissipation surface area but also induce surface phonon polaritons, enhancing absorption in specific wavelength ranges and increasing reflection and scattering [28,29]. The study of radiation cooling performance in organisms has inspired the design and fabrication of various biomimetic radiative cooling materials and devices [30,31,32,33].

In this study, we chose the *Cerambycini Latreille*, which inhabits tropical regions as the biological model. The hairy structures on the body of the *Cerambycini Latreille* were selected as the biomimetic prototype. We conducted a comprehensive investigation into the microstructure, major components, and distribution patterns of these hair structures, aiming to understand the factors that influence their thermal regulation performance. Utilizing the Effective Medium Theory and FDTD optical simulation analysis, the inherent connection between the microstructure of the hair on the body of the *Cerambycini Latreille* and its daytime radiative cooling characteristics was unveiled. This served as the theoretical foundation for the design and fabrication of biomimetic daytime radiative cooling devices. By employing alkali etching and template methods, a BRCF featuring a pyramid structure was successfully produced. This film demonstrates the ability to effectively reflect solar radiation and enhance absorption in the atmospheric window wavelength range. This work holds the potential to provide novel theoretical insights and technological support for the design and development of daytime radiative cooling devices. As a typical biological example of radiative cooling, the *Cerambycini Latreille* was first discovered in Asia in 1802 [34]. The primary habitat of the *Cerambycini Latreilleis* concentrated in tropical regions such as Southeast Asia, and it has also been found in some high-temperature active volcanic zones [35]. This is mainly due to its unique high-temperature adaptation ability [31].

## 2. Materials and Methods

### 2.1. Materials

Si (P-100), (P-110), and (P-111), and polycrystalline silicon, were purchased from Shanghai Suiying Photovoltaic Technology Co., Ltd. (Shanghai, China). Si (N-100), (N-110), and (N-111) were purchased from Harbin Tebo Technology Co., Ltd. (Harbin, China). Potassium hydroxide (purity 95%), isopropanol (A.R.), titanium dioxide particles (EP), and silica particles were purchased from Shanghai Aladdin Bio-Chem Technology Co., Ltd. (Shanghai, China). Deionized water (I) was purchased from Shanghai Bohu Biological Technology Co., Ltd. (Shanghai, China). Anhydrous ethanol (A.R.) was purchased from China National Pharmaceutical Group Chemical Reagent Co., Ltd. (Changchun, China). Ecoflex Silicone Rubber (Ecoflex, 00-35) was purchased from Smooth-on. Polydimethylsiloxane (PDMS) was purchased from Dow Corning Corporation. (Changchun, China).

### 2.2. Preparation of Templates by Alkaline Etching Method

First, the silicon wafers were sequentially cleaned using deionized water and anhydrous ethanol for 10 min each to remove surface impurities. A 10% potassium hydroxide solution was prepared and set aside. Then, according to different experimental designs, the silicon wafers were placed in sufficient amounts of the potassium hydroxide solution and placed in a thermostatic drying oven to maintain the experimental temperature. Various experimental schemes were designed to prepare appropriate pyramid structures. Six sets of each type of silicon wafer were prepared. The reactions were conducted at temperatures of 30 °C, 60 °C, and 90 °C, and the silicon wafers were removed and dried at 10 min, 30 min, 60 min, and 120 min of reaction time. The surface structure changes were observed under the SEM. Based on the etching experiments with different types of silicon wafers (Figure S2), it was found that only polycrystalline silicon had the potential to etch out pyramid or cone structures. Therefore, polycrystalline silicon wafers were chosen as the substrate for the biomimetic structural template, and the surface that closely resembled the optimized structure from the simulation was selected as the template for fabricating the BRCF.

### 2.3. Preparation of BRCF by Template Method

By etching the silicon wafers, appropriately etched polycrystalline silicon was chosen as the surface structure template. The next step involves fabricating the BRCF on the template. Due to the high emissivity of polydimethylsiloxane (PDMS) in the atmospheric window wavelength range, PDMS was chosen as the base material. Silicon dioxide (SiO_2_) particles were doped into the PDMS to enhance the emissivity in the atmospheric window wavelength range by utilizing the phenomenon of phonon polaritons resonance. However, the addition of SiO_2_ particles significantly reduces the flowability of PDMS and its stretchability. This reduction not only affects the replication of the silicon wafer surface structure but also makes it challenging to separate the BRCF from the silicon wafer. To address these issues, Ecoflex, which has better flowability, was chosen to replicate the silicon wafer surface structure. SiO_2_ particles were also doped into Ecoflex to enhance scattering in the solar spectrum. In addition to improved flowability, Ecoflex possesses strong stretchability, compensating for the drawback of PDMS’s difficulty in detaching from the silicon wafer. To illustrate the performance differences between Polydimethylsiloxane (PDMS) and Ecoflex, a tensile strength test was conducted as shown in Figure S3a,b. Ecoflex exhibited better stretchability, with its strain capacity being approximately 2.5 times that of PDMS for the same cross-sectional area. However, PDMS demonstrated superior mechanical properties, with a maximum load-bearing capacity of 8N under tension, whereas Ecoflex could withstand only 2N. Therefore, Ecoflex was selected as the first layer material for spin-coating onto the silicon wafer to better replicate the surface structure. Since the addition of particles significantly decreases the flowability of PDMS, titanium dioxide particles, which enhance solar spectral reflectance, were doped into Ecoflex to relieve the pressure on PDMS. After selecting the appropriate materials, the fabrication process of the BRCF was initiated. The fabrication process is illustrated in Figure S3c. Titanium dioxide particles were doped into Ecoflex at a 5% ratio, while silicon dioxide particles were doped into PDMS. After thoroughly mixing using a magnetic stirrer, the Ecoflex mixture was spin coated onto the silicon wafer’s etched surface. The coated wafer was then cured at 70 °C in a temperature-controlled oven for 30 min. Subsequently, the PDMS mixture was spin coated onto the cured Ecoflex layer and cured for 4 h at 70 °C. This process was repeated, ultimately forming a four-layer structure of Ecoflex-PDMS–Ecoflex-PDMS for the BRCF.

### 2.4. Analysis and Testing Methods

#### 2.4.1. Observation with Super Depth of Field Microscope (VHX-6000, KEYENCE, Tokyo, Japan)

- Use tweezers to hold the elytra’s hind part and rotate it along the direction of wing expansion to unfold the elytra and remove them by turning them upwards;
- Select a relatively flat area on the elytra and cut it into a 1 cm × 1 cm square for easy photography;
- Clean the cut square with acetone, ethanol, and deionized water using ultrasonic cleaning;
- After drying in an oven, place it under the Super Depth of Field Microscope for observation.

#### 2.4.2. Observation with SEM (JEOL Ltd., Tokyo, Japan)

The front-end processing procedure is similar to the process of observation with the Super Depth of Field Microscope. The difference is that in step b of the process, the sample needs to be cut into a strip measuring 10 mm × 1 mm so that it can be attached to the specimen holder. Due to the poor conductivity of dried biological samples, after step d processing, the biological sample attached to the specimen holder needs to undergo gold sputtering treatment using a plasma sputter coater. Subsequently, the sample, along with the specimen holder, is placed under the scanning electron microscope for observation.

#### 2.4.3. Sectioning with an Ultramicrotome

The front-end processing procedure is similar to the process of observing the *Cerambycini Latreille* under the Super Depth of Field Microscope. After step d, it is necessary to fix the elytra of the *Cerambycini Latreille*. The volume ratio of tissue to fixative is 1:25. In this experiment, 10% formaldehyde is used for fixation for 3 days, followed by 1 h of paraffin infiltration. Paraffin embedding is then performed to ensure complete integration of the tissue with paraffin. After embedding, a cold water bath is used to ensure rapid solidification of paraffin. Subsequently, slicing can be carried out using a microtome.

#### 2.4.4. EDS Testing

The sample preparation process is the same as the process for SEM. EDS is connected to the SEM, allowing energy-dispersive X-ray spectroscopy measurements to be conducted simultaneously during image acquisition.

#### 2.4.5. FTIR Testing

When utilizing a Fourier Transform Infrared Spectrometer to determine functional groups, KBr is used as a sample background. During the measurement process, the software automatically performs ten scans of the background or sample and averages the results to ensure accuracy. After subtracting the background spectrum, the sample’s spectral data are obtained.

### 2.5. Simulation Conditions

#### 2.5.1. The Simulation Conditions of the Primary Feature Structures

- Model Import: Due to modeling constraints, the model is imported at a scale of 1:0.0001 to achieve the actual size of the setae (in the order of 10 μm).
- Define Material Properties: Choose the refractive index property for configuration, set to the intrinsic refractive index of chitin, which is 1.56.
- Define Simulation Area and Boundary Conditions: When using symmetry boundaries, the simulation area boundary needs to maintain a distance of at least half a wavelength from the structure. Due to simulating non-periodic structures, here, we use Perfectly Matched Layer (PML) boundaries for absorption. Additionally, consider setting monitors along the z-axis direction for subsequent analysis. Therefore, the simulation area is set to 20 μm × 20 μm × 10 μm, with an automatic non-uniform grid setting and second-order accuracy.
- Set the grid step sizes in the x, y, and z directions to 0.4 μm, 0.4 μm, and 5 μm, respectively. Ensure that the grid boundaries in all three directions are greater than 0.2 μm beyond the structure.
- Set Light Source: After reaching the Earth, solar radiation can be approximated as parallel light. Therefore, set the light source as a plane wave, with the incident direction along the negative y-axis. The distance of the light source from the structure is more than half a wavelength. For simulating the solar spectral range, set the distance to 1.25 μm, and for simulating the atmospheric window range, set the distance to 6.5 μm.
- Set Monitors: In the y-direction, place frequency-domain power monitors both above and below the structure to obtain reflectance and transmittance data. The direction is along the xz plane. To capture the vector plots, electric field variations, and light propagation paths during the simulation, set frequency-domain profile monitors and movie monitors along the xy plane.
- Run the simulation.

#### 2.5.2. The Simulation Conditions of the Secondary Biological Feature Structures

- Model Creation: After creating a rectangular base, the triangular structure was directly imported using the grating option. The model for the semi-circular structure was established by combining cylindrical and rectangular structures.
- Define Material Properties: Choose the refractive index property for configuration, set to the intrinsic refractive index of chitin, which is 1.56.
- Define Simulation Domain and Boundary Conditions: For the simulation of the secondary structure with periodicity, maintain a boundary distance of at least half a wavelength when using symmetric boundaries. Use periodic boundaries in the x-direction and perfectly matched layer (PML) boundaries in the y-direction. Similar to the primary structure, consider setting monitors along the z-axis direction. As the secondary structure will be simulated for both base lengths/diameters of 1.5 µm and 2 µm, set the simulation domain to 4 µm × 6 µm × 1 µm and 3 µm × 5 µm × 1 µm for the two sizes. Utilize an automatic non-uniform grid setting with second-order accuracy.
- Grid Partitioning: 0.1 µm in the x-direction, 0.1 µm in the y-direction, and 1 µm in the z-direction.
- Setting the Light: The light source is configured as a plane wave with its incident direction along the negative y-axis. The distance between the light source and the structure is set to be at least half a wavelength. For simulating the solar spectral range, the distance is set to 1.25 µm, and for simulating the atmospheric window range, the distance is set to 6.5 µm.
- Setting Monitors: In the y-direction, frequency domain power monitors are placed both above and below the structure to obtain reflectance and transmittance data. These monitors are oriented along the xz plane. To obtain Poisson vector plots, electric field profiles, and light propagation paths during the simulation process, frequency domain profile monitors and movie monitors are positioned along the xy plane.
- Run the simulation.

## 3. Results and Discussion

### 3.1. Radiation Cooling Characteristics and Characterization of Cerambycini Latreille

**Figure 1:**
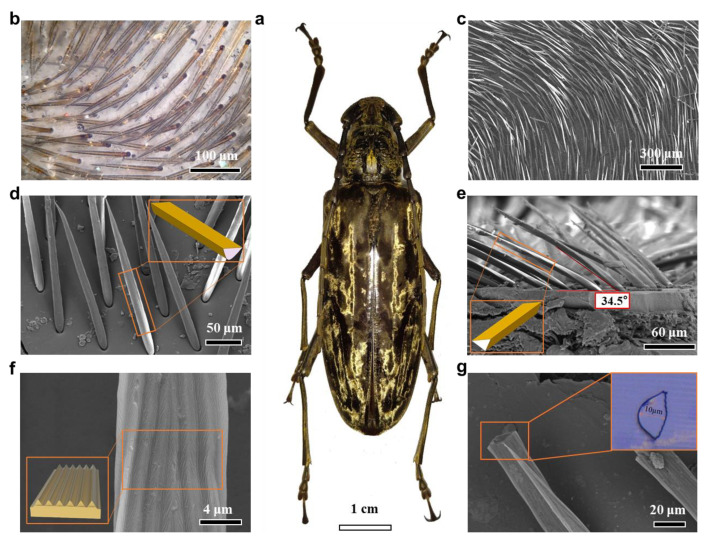
The *Cerambycini Latreille* and its hair morphology characteristics. (**a**) The overall morphology of the *Cerambycini Latreille*. (**b**) Super Depth of Field Microscope image of the beetle hair distribution. (**c**) SEM image of the beetle hair distribution. (**d**) SEM morphology image of a single strand of beetle hair. (**e**) Side view SEM image of a single strand of beetle hair. (**f**) SEM image of the secondary structure on the surface of the hair. (**g**) Cross-sectional SEM image of the hair.

Previous research has demonstrated that the thermal regulation ability of the *Cerambycini Latreilleis* mainly attributed to its body’s triangular-shaped setae. This layer of densely covered setae is present on the surface of the Giant Longhorn Beetle’s body, as shown in Figure 1a. Subsequently, the distribution and morphology of setae on the surface of the *Cerambycini Latreille* were observed using super-depth microscopy and scanning electron microscopy (SEM). From the captured overall image of the *Cerambycini Latreille* (Figure 1b), it is clearly visible that its body is covered with a dense layer of golden setae. Moreover, the intensity of the golden reflection on its body surface varies when observed from different directions. This indicates that the structure of individual setae can achieve strong reflection in the visible light spectrum. Through observations using SEM, as shown in Figure 1c–g, it can be observed that the setae grow at an angle on the surface of the *Cerambycini Latreille*, and their growth directions are relatively chaotic, mostly forming a vortex-like pattern. However, due to their dense growth, the entire body surface is almost completely covered. Further observation of individual setae reveals that their length is approximately 250 µm, with an overall width ranging from 10 to 20 μm. The 20 μm width region constitutes around 70% of the total length of the setae (excluding the tip). The individual setae exhibit a tapering shape, narrower at the top and wider at the base, resembling a semi-conical structure. This morphology is referred to as the primary structure. As shown in Figure 1e, it can be observed that the angle of inclination between the setae and the body surface is approximately 34.5°. Further observation of the specific morphology of individual setae reveals the presence of striped protuberances on the primary structure. These protuberances grow along the longitudinal direction of the setae, with a lateral width of approximately 1.5 to 2 μm. This is referred to as the secondary structure. Finally, observations were made of the cross section of the setae. After the setae were sliced using an ultra-thin sectioning machine and then observed using SEM, it was found that the outer surface was wider, while the inner surface curved inward into a semi-circular shape. The overall morphology is wider on the outside and narrower on the inside, providing a foundation for the subsequent construction of biomimetic feature structural models.

In previous studies, researchers have already demonstrated that the setae possess strong visible light reflection ability and mid-infrared emission ability. To investigate the influence of materials on their temperature regulation capability, an Energy Dispersive Spectrometer (EDS) was utilized to quantitatively describe the elemental composition ratios, and a Fourier Transform Infrared Spectrometer (FTIR) was used to determine the major functional groups of the setae, aiming to identify their primary constituents. The energy dispersive spectroscopy measurements, as shown in Figure S1a, revealed that the surface composition of the setae primarily includes elements such as carbon (C), nitrogen (N), and oxygen (O). The mass percentages of carbon, nitrogen, and oxygen are approximately 59.8%, 10.24%, and 28.55%, respectively, while silicon (Si), potassium (K), and calcium (Ca) each constitute less than 1%, and can be considered negligible. After determining the elemental composition and their respective proportions in the setae of the *Cerambycini Latreille*, further analysis of the primary constituents of the setae can be conducted by combining the measurements of functional groups. The Fourier Transform Infrared Spectroscopy (FTIR) results of the *Cerambycini Latreille* setae are shown in Figure S1b. The characteristic peaks at 1550 cm^−1^ and 1680 cm^−1^ correspond to the amide bands II and I of the amide functional groups, respectively. The former is attributed to the C=O stretching vibration coupled with N-H bending, while the latter arises from the C-N stretching vibration coupled with N-H bending. These two characteristic peaks indicate the presence of chitin and protein components in the setae. The weak absorption peaks at 2977 cm^−1^, 2916 cm^−1^, and 2873 cm^−1^ correspond to the symmetric and antisymmetric stretching vibrations of C-H bonds. The broad absorption peaks at 3432 cm^−1^ and 3256 cm^−1^ correspond to the stretching vibrations of hydroxyl (OH) groups and N-H bonds, respectively. Considering the above results collectively, it can be concluded that the *Cerambycini Latreille* setae are primarily composed of chitin.

There are primarily three modes of heat transfer: conduction, radiation, and convection. In the field of radiative cooling, the thermal regulation ability of organisms is mainly discussed in terms of thermal radiation. Thermal radiation refers to the emission of energy in the form of electromagnetic waves, and an object’s ability to absorb thermal radiation is primarily influenced by its material, temperature, and structure. At room temperature (300 K), an object’s material and structure determine its reflectance and transmittance in different wavelength ranges. According to the effective medium theory, an analysis is conducted on the structures of two different size levels in the *Cerambycini Latreille* setae.

When the isotropic periodic structure of a grating has a much smaller period than the wavelength, generally around 1/8 to 1/10 of the wavelength, it is suitable to apply the effective medium theory for calculations. For the primary structure, within the wavelengths of interest, its larger period allows for an explanation using specular reflection theory for visible and near-infrared light, suggesting the potential for strong reflection in these ranges. As for the secondary structure, within the wavelengths of interest, its period approaches 1/8 to 1/10 of the wavelength in certain wavelength bands. This allows for calculations using effective medium theory and simulation modeling.

### 3.2. Biomimetic Design of Primary Feature Structures

Based on the characteristic parameters of the setae structure obtained from morphological characterization, an appropriate three-dimensional model is established. The theoretical analysis is validated using FDTD-Solutions simulation software (Lumerical version: 8.15.736), and the three-dimensional model is optimized based on simulation results to establish an improved radiative cooling structural model. According to the observed morphology, the cross section of the primary structure of the *Cerambycini Latreille* setae exhibits a characteristic of being wider at the top and narrower at the bottom, presenting an overall semi-circular shape (Figure 1d), or it can be further simplified into an isosceles triangle structure (Figure 1e). Using the dimension data obtained from characterization, these two models are incorporated and simulated using FDTD Solutions software to obtain their reflectance, transmittance in the solar spectral range, and emissivity in atmospheric window wavelengths. The simulation software is utilized to observe the propagation characteristics of electromagnetic waves at different wavelengths on the cross section and to analyze the results to select a more suitable structure. With the movement of the *Cerambycini Latreille*, the angle of sunlight incident on the setae also varies. Therefore, the choice is made to have light incident along the cross section, and a three-dimensional structural simulation is selected to achieve more accurate simulation results. In order to investigate the influence of structural shape on solar spectral reflection and atmospheric window emissions, a rectangular cross-sectional structure is set as a control group. The simulation conditions are established in Section 2.5.1.

**Figure 2:**
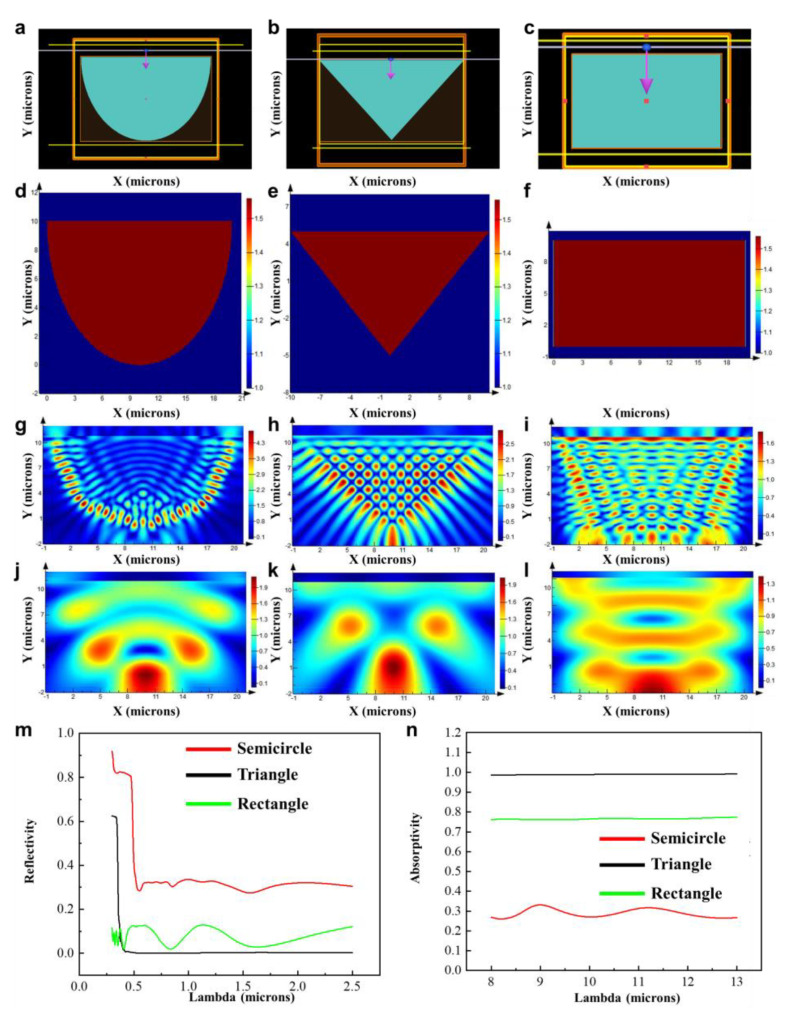
Simulation and testing of primary structure of *Cerambycini Latreille.* (**a**) Schematic of biomimetic conditions for semi-circular structure. (**b**) Schematic of biomimetic conditions for triangular structure. (**c**) Schematic of biomimetic conditions for rectangular structure. (**d**) Illustration of cross section and structural refractive index of semi-circular model. (**e**) Illustration of cross section and structural refractive index of triangular model. (**f**) Illustration of cross section and structural refractive index of rectangular model. (**g**) Electric field distribution of semi-circular cross section structure in solar radiation band. (**h**) Electric field distribution of triangular cross-section structure in solar radiation band. (**i**) Electric field distribution of rectangular cross-section structure in solar radiation band. (**j**) Electric field distribution of semi-circular cross-section structure in atmospheric window band. (**k**) Electric field distribution of triangular cross-section structure in atmospheric window band. (**l**) Electric field distribution of rectangular cross-section structure in atmospheric window band. (**m**) Simulation results of solar spectral band reflectance for three structures. (**n**) Simulation results of atmospheric window band absorption for three structures.

Figure 2a–c depict the conceptual illustration of FDTD biomimetic conditions for the primary structure. Figure 2d–f show the cross section of the model and the refractive index schematic. Figure 2g,j display the electric field distribution in the solar spectral range for the semi-circular cross-sectional structure and the atmospheric window range, respectively. Similarly, Figure 2h,k depict the electric field distribution in the solar spectral range and the atmospheric window range for the triangular cross-sectional structure. Figure 2i,l illustrate the electric field distribution in the solar spectral range and the atmospheric window range for the rectangular cross-sectional structure. For a clearer comparison of the changes in the three structures’ light absorption, the results have been graphically presented in Figure 2m,n. Comparing the data from the three structures, it can be observed that (a) the semi-circular cross-sectional structure has a significantly higher reflectance in the solar spectral range compared to the triangular and rectangular cross-sectional structures. As evident from the electric field distribution shown in Figure 2g,h, the electric field intensity at the interface of the semi-circular cross-sectional structure is twice that of the triangular cross-sectional structure. When simplifying the model, it can be seen that the semi-circular cross-sectional structure closely resembles the original characteristics of the setae, aligning with the macroscopic observation that the Giant Longhorn Beetle’s body surface strongly reflects visible light. (b) The triangular cross-sectional structure exhibits significantly higher absorbance in the atmospheric window range compared to the semi-circular and rectangular cross-sectional structures. This suggests that, under the same material conditions, the triangular structure has a strong promoting effect on absorption in the atmospheric window range. This effect is likely attributed to the gradient refractive index caused by the structural gradient variations. Based on the aforementioned points, it is believed that the triangular cross-sectional structure holds a stronger value for radiative cooling applications. To address its lower reflectance in the solar spectral range, optimization of the dimensions and shape of the triangular cross-sectional structure will be pursued to enhance reflectance in this range. Additionally, in the practical fabrication of daytime radiative coolers, particular emphasis will be placed on utilizing materials to enhance reflectance in the solar spectral range.

**Figure 3:**
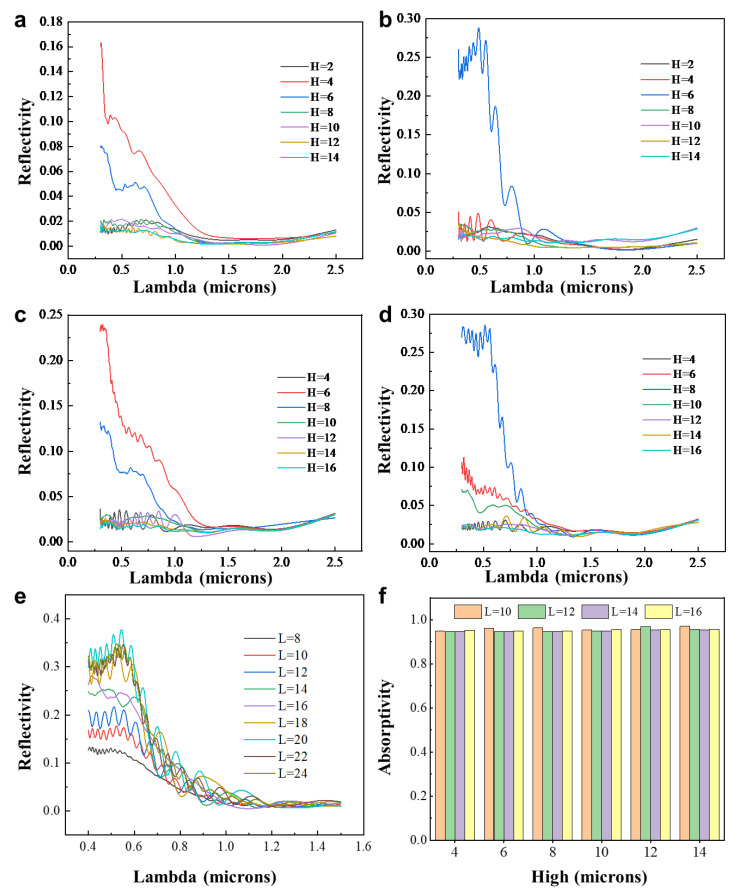
Solar spectral reflectance of models with different base widths and heights. (**a**) Solar spectral reflectance of the model with a base width of 10 μm. (**b**) Solar spectral reflectance of the model with a base width of 12 μm. (**c**) Solar spectral reflectance of the model with a base width of 14 μm. (**d**) Solar spectral reflectance of the model with a base width of 16 μm. (**e**) Simulated reflectance of isosceles triangular cross-section structures with different base widths. (**f**) Simulated data of atmospheric window absorption for triangular cross-section structures with different base widths and heights.

Next, we will explore suitable dimensions for the triangular cross-sectional structure to achieve higher reflectance in the solar spectral range and higher emission in the atmospheric window range. Different sizes and shapes of triangular structures will be designed by controlling their base length and height. Through simulation modeling, we will obtain data on the reflectance in the solar spectral range and the emission in the atmospheric window range for each design. Initially, we will select four triangular cross-sectional structures with base lengths of 10, 12, 14, and 16, each with varying heights, in order to investigate the relationship between shape and size variations and the reflectance in the solar spectral range as well as the emission in the atmospheric window range. The simulation results are presented in Figure 3. From the simulation results, the following conclusions can be drawn: (a) Keeping the base length constant, as the height approaches half of the base length, the reflectance in the ultraviolet and visible regions increases. There is little change in the infrared region. (b) When the height/base length ratio approaches 1/2, increasing the base length leads to higher reflectance in the ultraviolet and visible regions, with little change in the infrared region. (c) Longer base lengths result in a wider range of wavelengths affected, indicating a clear correlation between model size and wavelength. To find the dimensions with the highest reflectance, while maintaining a height/base length ratio of 1/2, the size of the triangular structure is further increased. The reflectance simulation data in the solar spectral range for different base lengths (ranging from 8 μm to 24 μm with increments of one integer) are obtained, as shown in Figure 3e. From the simulation results, it can be observed that the reflectance begins to stabilize as the base length reaches around 20 μm, which aligns with the observed size of the primary structure of the setae from biological morphology observations.

Furthermore, an exploration of the atmospheric window absorption data for different-sized structures was conducted, and the simulation test results are shown in Figure 3f. The results indicate that regardless of the structure size, the absorption remains around 0.93–0.95. Consequently, it can be concluded that for chitin material, the atmospheric window absorption of the ten-micrometer-scale triangular structure remains relatively consistent. In summary, the optimal choice for a primary visible light reflection structure is an isosceles triangular cross-sectional structure with a base length of 20 μm and a height of 10 μm.

### 3.3. Biomimetic Design of Secondary Biological Feature Structures

**Figure 4:**
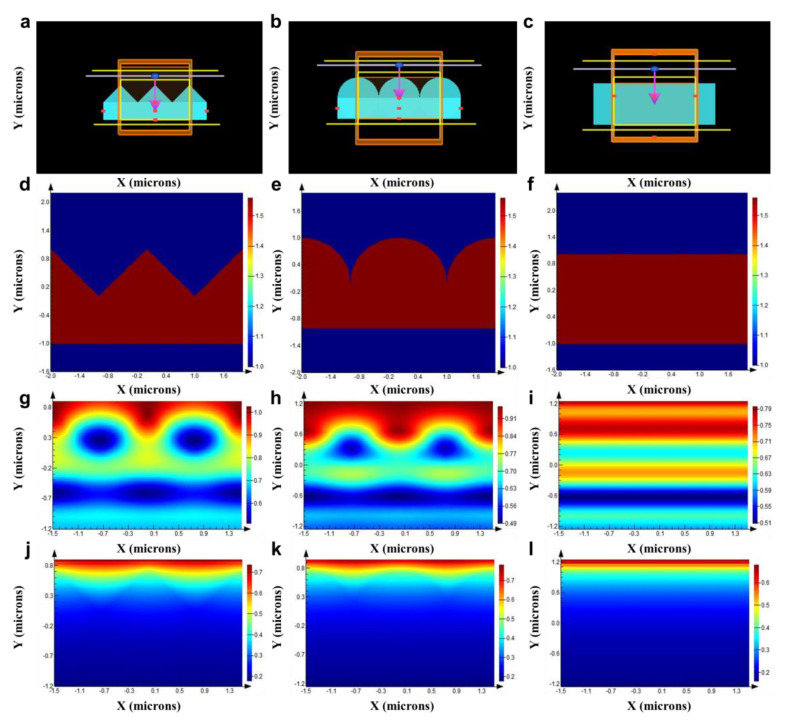
Second-order structure FDTD simulation schematic. (**a**) Triangular structure biomimetic condition setting diagram. (**b**) Semi-circular structure biomimetic condition setting diagram. (**c**) Rectangular structure biomimetic condition setting diagram. (**d**) Triangular model cross-section and structural refractive index schematic. (**e**) Semi-circular model cross-section and structural refractive index schematic. (**f**) Rectangular model cross-section and structural refractive index schematic. (**g**) Electric field distribution in the solar radiation band of the triangular cross-section structure. (**h**) Electric field distribution in the solar radiation band of the semi-circular cross-section structure. (**i**) Electric field distribution in the solar radiation band of the rectangular cross-section structure. (**j**) Electric field distribution in the atmospheric window band of the triangular cross-section structure. (**k**) Electric field distribution in the atmospheric window band of the semi-circular cross-section structure. (**l**) Electric field distribution in the atmospheric window band of the rectangular cross-section structure.

**Figure 5:**
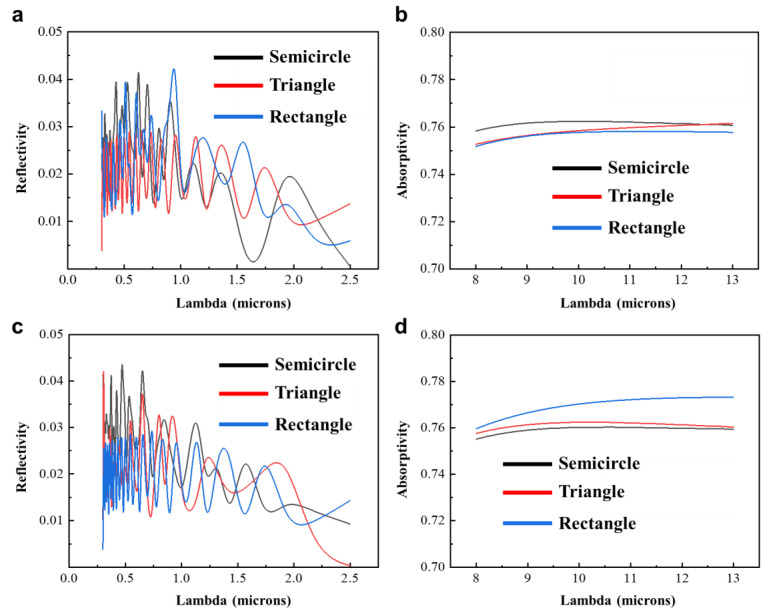
Simulated results of the secondary structure. (**a**) Simulated results of solar spectrum reflectance for the three structures with a protrusion height of 0.75 µm. (**b**) Simulated results of atmospheric window band absorption for the three structures with a protrusion height of 0.75 µm. (**c**) Simulated results of solar spectrum reflectance for the three structures with a protrusion height of 1 µm. (**d**) Simulated results of atmospheric window band absorption for the three structures with a protrusion height of 1 µm.

Subsequently, based on the observed surface morphology of the secondary structure, simplified models of the *Cerambycini Latreillesetae*’s secondary structure were established. Similarly, three simplified models were created: semi-circular protrusion, triangular protrusion, and rectangular protrusion. The cross-sectional morphologies of the three structures are depicted in Figure 4a–c. To avoid accidental results, two sizes of structures were established: base length/diameter of 1.5 µm and 2 µm, with heights of 0.75 µm and 1 µm. The simulation design for the secondary structure differs from that of the primary structure, and the settings for simulating the secondary structure are as follows:

Figure 4a–c depict the schematic diagram of the first-level structure FDTD biomimetic conditions, while Figure 4d–f show the sectional view and structural refractive index diagram of the model. Figure 4g,j illustrate the electric field distribution of the triangular cross-section structure in the solar radiation band (Figure 4g) and the atmospheric window band (Figure 4j). Figure 4h,k display the electric field distribution of the semi-circular cross-section structure in the solar radiation band (Figure 2h) and the atmospheric window band (Figure 2k). Figure 2i,l represent the electric field distribution of the rectangular cross-section structure in the solar radiation band (Figure 2i) and the atmospheric window band (Figure 2l). Based on the simulation results, the reflectance data for the solar spectrum and the absorptance data for the atmospheric window band were obtained for the three types of structures. Figure 5a–d present simulation results for the three different cross-sectional models. By comparing the various data, it can be concluded that the influence of the two structures on the reflection in the solar spectrum and emission in the atmospheric window band is minor at the scale of 1.5–2 µm and can be neglected.

### 3.4. Biomimetic Mapping Structure and Simulation

Based on the simulation results, it is evident that the first-level structure exhibits the strongest enhancement effect on daytime radiative cooling. Not only does it effectively enhance absorption in the atmospheric window band, but it also moderately enhances visible light reflection. On the other hand, the enhancement effect of the second-level structure is not as pronounced. Therefore, following the principle of similarity, the first-level triangular structure was further simplified into a pyramid structure. Simulation and verification were conducted to assess whether the pyramid structure meets the requirements for reflectance in the atmospheric window band and solar spectrum. Furthermore, the first-level structure was simplified into an array structure, necessitating the establishment of new simulation conditions. Thus, the following settings were applied for both the array structure and the blank control simulation:

- Model Establishment: The pyramid structure with a base edge length of 10 μm was directly created using the “structure” command. Since the conical structure cannot be directly built, it was externally designed and then imported. Following this, a rectangle base with the same refractive index was constructed beneath it.
- Define Material Properties: Choose the refractive index attribute and set it to the inherent refractive index of chitin, which is 1.56.
- Define Simulation Domain and Boundary Conditions: When setting the simulation domain, reserve a space of 13 μm above the structure for placing the light source and reflectance monitors. Use a perfectly matched layer (PML) boundary for absorption, and set the grid to an automatic non-uniform grid with second-order accuracy.
- Grid Partitioning: The grid step sizes are set as 0.1 µm in the x, y, and z directions. The grid boundaries are not restricted anymore and should match the size of the structure.
- Setting the Light Source: The light source is set as a plane wave, with the incident direction along the negative y-axis. The distance of the light source from the structure is more than half a wavelength. For simulating the solar spectrum range, the distance is set at 1.25 µm. For simulating the atmospheric window spectrum range, the distance is set at 6.5 µm.
- Setting Monitors: In the z-direction, frequency domain power monitors are placed above and below the structure to obtain reflectance and transmittance data. The direction is along the xy-plane. To capture the Poisson vector plot, electric field variation plot, and light propagation path during the simulation, frequency domain profile monitors and movie monitors are set along the xz-plane.
- Run the simulation.

**Figure 6:**
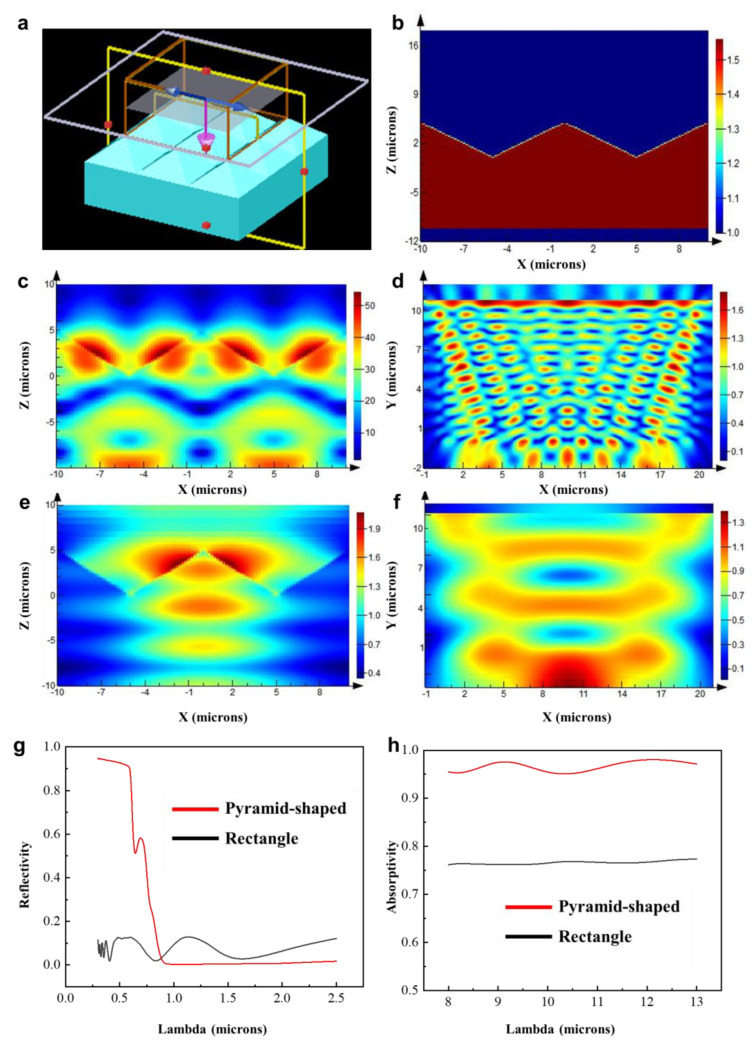
Simulated results of biomimetic pyramid structure. (**a**) Simulation setup of pyramid structure. (**b**) Structural refractive index schematic. (**c**) Electric field distribution in solar spectrum of pyramid protrusion structure. (**d**) Electric field distribution in solar spectrum for comparative structure. (**e**) Electric field distribution in atmospheric window band of pyramid protrusion structure. (**f**) Electric field distribution in atmospheric window band for comparative structure. (**g**) Simulated absorption rates in atmospheric window band for both structures. (**h**) Simulated reflectance rates in solar spectrum for both structures.

Figure 6a,b depict the schematic of FDTD biomimetic conditions for the first-level structure. Figure 6c,e show the electromagnetic field distribution in the solar spectrum range (Figure 6c) and the atmospheric window range (Figure 6e) for the biomimetic pyramid protrusion structure. Figure 6d,f illustrate the electromagnetic field distribution in the solar spectrum range (Figure 6d) and the atmospheric window range (Figure 6f) for the control group rectangular section structure. Based on the simulation results, the reflectance data in the solar spectrum range and the absorptance data in the atmospheric window range for both structures are obtained, as shown in Figure 6g,h. By comparing the simulated data of the two structures, the following conclusions can be drawn: (a) Compared to the structureless model, the pyramid protrusion can effectively increase the reflectance in the visible light spectrum, while the reflectance in the infrared spectrum is nearly zero. (b) Compared to the structureless model, the pyramid protrusion can significantly enhance the absorptance in the atmospheric window range, with an increase of approximately 20%.

### 3.5. Characterization and Testing of the BRCF

**Figure 7:**
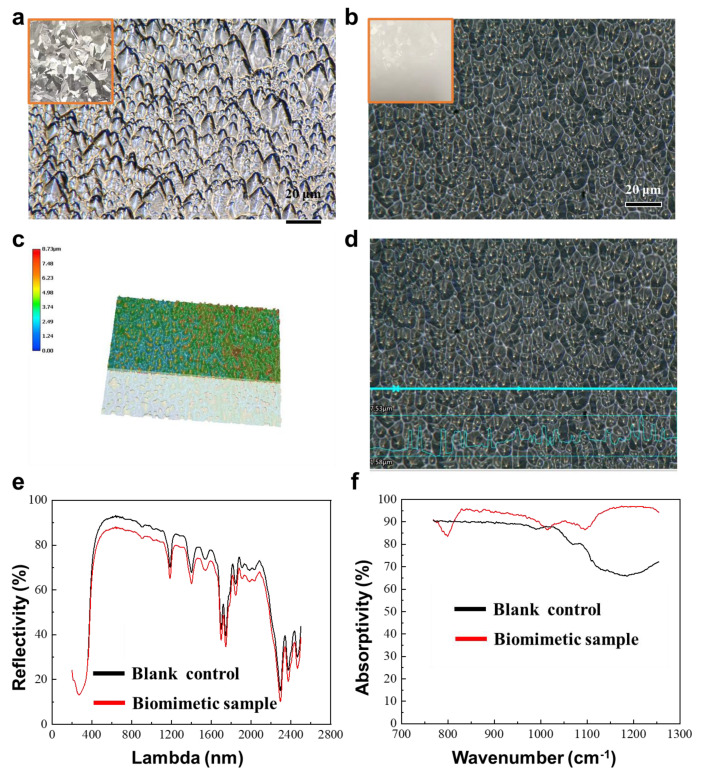
Characterization and performance testing of BRCF. (**a**) Silicon wafer morphology and surface structure. (**b**) BRCF and its surface structure. (**c**) SEM morphology image of BRCF. (**d**) SEM structural morphology image of the BRCF. (**e**) Solar spectral reflectance of the BRCF. (**f**) Atmospheric window absorption rate of the BRCF.

After the preparation of the daytime BRCF and its surface-structured silicon template, in order to characterize the structural features and daytime radiative cooling properties, the surfaces of the BRCF and the silicon template were observed using a confocal microscope. The solar spectral reflectance of the BRCF was measured using a UV-visible spectrophotometer, and the atmospheric window emission was measured using an infrared spectrometer. The etched surface of the silicon wafer is shown in Figure 7a, and this surface structure was transferred to the Ecoflex mixture surface, resulting in irregular pyramid-shaped structures, as shown in Figure 7b. These replicated pyramid structures have dimensions ranging from 1 to 15 μm, covering both the first and second-level structures of the beetle’s hair. Through confocal microscopy imaging, as shown in Figure 7c,d, it can be observed that the heights of the structures vary. The maximum height difference of the pyramid-shaped structures is approximately 5–6 μm, while the minimum height difference is about 1–2 μm. The height-to-diameter ratio of the cone-shaped structures is approximately 1/2. The most intuitive performance indicator of daytime radiative cooling devices is the cooling effect under sunlight. Solar spectral reflectance and atmospheric window emissivity are the main performance metrics for evaluating daytime radiative cooling devices. However, measuring this indicator during winter temperature changes is challenging. Therefore, the solar spectral reflectance and atmospheric window emissivity are used as measurement indicators to analyze the performance enhancement brought about by the structural design. As shown in Figure 7e, the daytime BRCF can achieve an emissivity of over 70% in most of the solar spectral range. This is attributed to the effective scattering of sunlight by the titanium dioxide particles doped in the BRCF. However, it is worth noting that the radiative cooling film without structures has a higher reflectance than the BRCF. This is because the pyramid-shaped structures play a role in light trapping in the visible and near-infrared scales, making it difficult for light to escape. From Figure 7f, it can be observed that the emissivity of the atmospheric window region for the BRCF with surface structures is significantly higher than that of the film without surface structures. Especially in the 8–10 micrometer range, the enhancement in absorptivity can reach over 30%. This indicates that the fabricated biomimetic surface enhances the absorption in the atmospheric window region.

## 4. Conclusions

Addressing the challenges of surface structure design and the complexity and cost of fabrication in daytime radiative cooling technology, a biomimetic approach was adopted. By characterizing the surface structure of the Hercules beetle’s setae, simplified models of different hierarchical levels of the setae structure were established. Through simulation and systematic analysis, the impact of these simplified models on the performance of daytime radiative cooling was studied. Further simplification led to the design of a biomimetic radiative cooling structure that could be easily fabricated, and its feasibility was verified through simulation. Based on this, an irregular inverted pyramid structure was fabricated on a silicon wafer surface using alkali etching as a template for producing the BRCF structure. Combining spin-coating processes, a BRCF with pyramid structures was successfully manufactured. This film effectively reflected solar radiation (70%) and enhanced absorption in the atmospheric window region (30%). This research provides new development insights into biomimetic radiative cooling materials and structures. This will be applied to surfaces such as glass architecture and automotive glass. It offers a reference for the future development of multifunctional radiative cooling materials, which are progressively moving towards multifunctionality, intelligence, composite functionality, environmental adaptability, high durability, and a wide range of applications in radiative cooling, thus contributing to a promising and versatile future for radiative cooling materials.

## Data Availability

Data are contained within the article.

## Supplementary Materials

The following supporting information can be downloaded at: https://www.mdpi.com/article/10.3390/biomimetics9010034/s1, Figure S1. Composition analysis of *Cerambycini Latreille* hair. (a) EDS test image of the *Cerambycini Latreille* hair. (b) FTIR test image of the *Cerambycini Latreille* hair. Figure S2. Surface morphology of template prepared by silicon wafer alkaline etching method. (A) Surface morphology changes of P-100 silicon wafers under different reaction conditions. (B) Surface morphology changes of P-110 silicon wafers under different reaction conditions. (C) Surface morphology changes of P-111 silicon wafers under different reaction conditions. (D) Surface morphology changes of N-100 silicon wafers under different reaction conditions. (E) Surface morphology changes of N-110 silicon wafers under different reaction conditions. (F) Surface morphology changes of N-111 silicon wafers under different reaction conditions. (G) Surface morphology changes of polycrystalline silicon wafers under different reaction conditions. Figure S3. Fabrication process of the BRCF. (a) The force–displacement curve of Ecoflex. (b) The force–displacement curve of PDMS. c) Fabrication process diagram.
