# Supplementary material for: A Study on the Radiation Cooling Characteristics of Cerambycini Latreille
Source: Biomimetics (Basel). 2024 Jan 4;9(1):34. doi: 10.3390/biomimetics9010034 (PMC10813328; doi:10.3390/biomimetics9010034)
Supplement: Supplementary file 1 [file biomimetics-09-00034-s001.zip › biomimetics-2635566-supplementary.pdf]

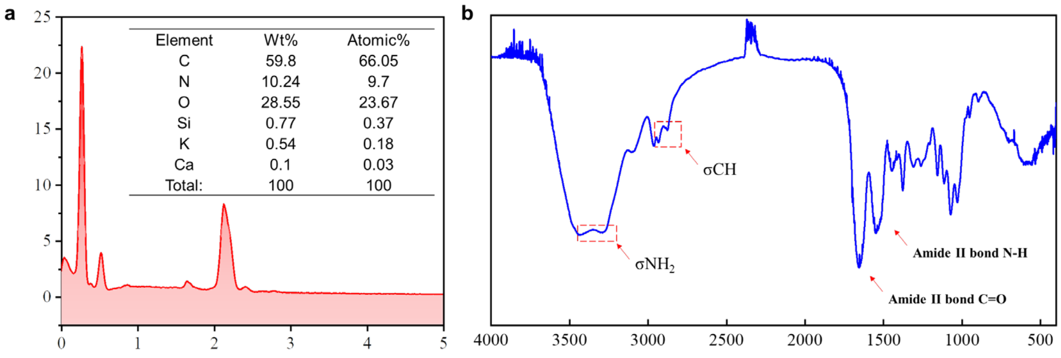

Figure S1. Composition analysis of Cer*ambycini Latreille* hair.

(a) EDS test image of the *Cerambycini Latreille* hair. (**b**) FTIR test image of the *Cerambycini Latreille* hair.

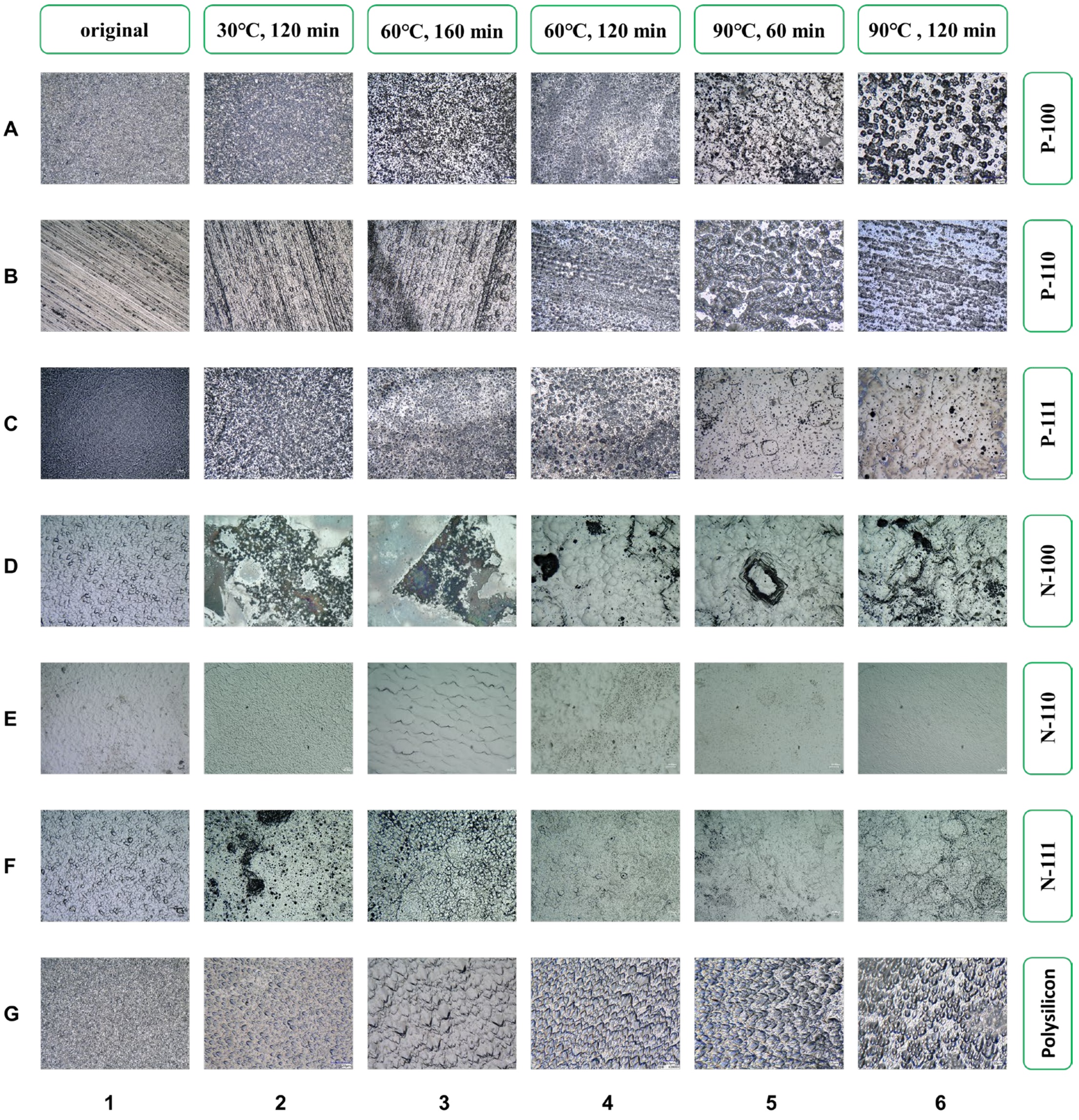

Figure S2. Surface morphology of template prepared by silicon wafer alkaline etching method.

A) Surface morphology changes of P-100 silicon wafers under different reaction conditions. B) Surface morphology changes of P -110 silicon wafers under different reaction conditions. C) Surface morphology changes of P-111 silicon wafers under different reaction conditions. D) Surface morphology changes of N-100 silicon wafers under different reaction conditions. E) Surface morphology changes of N-110 silicon wafers under different reaction conditions. F) Surface morphology changes of N-111 silicon wafers under different reaction conditions. G) Surface morphology changes of polycrystalline silicon wafers under different reaction conditions.

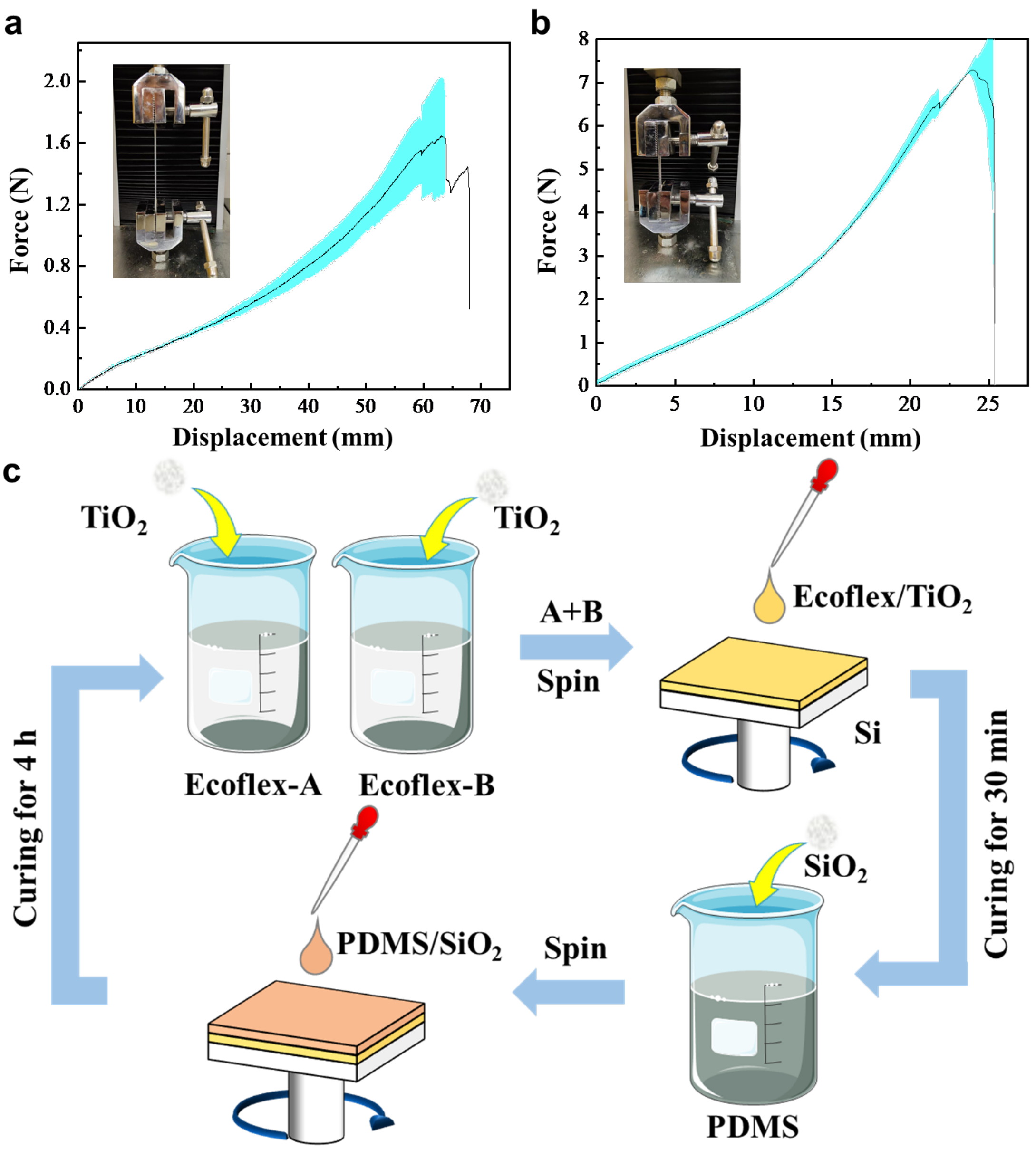

Figure S3. Fabrication process of the BRCF.

a) The force–displacement curve of Ecoflex. b) The force–displacement curve of PDMS. c) Fabrication process diagram.
